# Repellent activity against *Anopheles gambiae* of the leaves of nesting trees in the Sebitoli chimpanzee community of Kibale National Park, Uganda

**DOI:** 10.1186/s12936-022-04291-7

**Published:** 2022-09-27

**Authors:** Camille Lacroux, Emmanuelle Pouydebat, Marie Rossignol, Sophie Durand, Alfred Aleeje, Edward Asalu, Fabrice Chandre, Sabrina Krief

**Affiliations:** 1 UMR 7206 CNRS/MNHN/P7, Eco-Anthropologie, Museum National d’Histoire Naturelle, Musée de L’Homme, 17 place du Trocadéro, 75116 Paris, France; 2 UMR 7179 CNRS/MNHN, Mécanismes Adaptatifs Et Evolution, Museum National d’Histoire Naturelle, 57 rue Cuvier, 75231 Paris, France; 3 Sebitoli Chimpanzee Project, Great Apes Conservation Project, Kibale National Park, Fort Portal, Uganda; 4 La Phocéenne de Cosmétique, ZA Les Roquassiers, 174 Rue de la Forge, 13300 Salon-de-Provence, France; 5 Maladies Infectieuses et Vecteurs: Ecologie, Génétique, Evolution Et Contrôle, Institut de Recherche Et Développement, UMR MIVEGEC IRD/CNRS/Montpellier University, 911 avenue Agropolis, 34394 Montpellier Cedex 5, France; 6 Uganda Wildlife Authority, Kibale National Park, Fort Portal, Uganda

**Keywords:** Essential oil, Self-medication, *Anopheles gambiae*, *Pan troglodytes schweinfurthii*, Sleeping platform

## Abstract

**Background:**

Every evening, chimpanzees (*Pan troglodytes*) build a sleeping platform so called “nest” by intertwining branches of tree. Most of chimpanzees’ communities studied have a preference for tree species in which they nest. As female mosquitoes are feeding on the blood of their host at nighttime, chimpanzees may prevent being disturbed and bitten by mosquitoes by selecting tree species having properties to repel them.

**Methods:**

To test the hypothesis that chimpanzees choose tree species for their aromatic properties, data related to 1,081 nesting trees built between 2017 and 2019 in the Sebitoli community of Kibale National Park (Uganda) were analysed. The 10 most used trees were compared to the 10 most common trees in the habitat that were not preferred for nesting. Leaves from the 20 trees species were collected and hydro-distillated to obtain essential oils and one of the by-products for behavioural bioassays against females of the African mosquito, *Anopheles gambiae*.

**Results:**

Sebitoli chimpanzees showed tree preferences: 10 species correspond to more than 80% of the nesting trees. Out of the essential oil obtained from the 10 nesting trees, 7 extracts for at least one concentration tested showed spatial repellency, 7 were irritant by contact and none were toxic. In the other hand, for the abundant trees in their habitat not used by chimpanzees, only 3 were repellent and 5 irritants.

**Discussion and conclusion:**

This study contributes to evidence that chimpanzees, to avoid annoying mosquitoes, may select their nesting trees according to their repellent properties (linked to chemical parameters), a potential inspiration for human health.

**Supplementary Information:**

The online version contains supplementary material available at 10.1186/s12936-022-04291-7.

## Background

Every evening, all great apes—the seven species of the genus *Pan, Gorilla* and *Pongo—*build sleeping platforms commonly called ‘nests’ in which they will spend the night [1]. They intertwine branches or stems and foliage to build a construction which is mostly in trees but might also be on the ground for gorillas [2, 3]. The primary function of this ape shelters is to provide a stable and comfortable support to sleep. Additional functions have been proposed: (i) arboreal nests may reduce the incidence of night predation [4], (ii) a layer of insulation may help thermoregulation [5, 6], and (iii) it may provide a physical or chemical barrier against pathogens or parasites [5–7]. The nest height and the location of the nesting site may also provide some advantage to prevent insects bites [8]. Such features might be advantageous to avoid disease transmission because night is a key period when female mosquitoes take their blood meal and may be vectors of disease when infected.

These hypotheses focused on the function of the nest itself or the reasons behind arboreal versus terrestrial nests. Interestingly, populations of chimpanzees across all the sites studied appear to select particular tree species for nesting. This behaviour raises questions behind the choice of the tree species itself. Two major frameworks have been expressed to explain tree species preference: biomechanical (comfort) and/or biochemical (repellent) reasons. The most preferred nesting tree of the chimpanzee community from the Toro-Semliki Wildlife Reserve (Uganda), *Cynometra alexandri* appeared to be more comfortable by offering firmer and more stable support for the nest with thick foliage [9]. Hypothetically, apes may also prefer trees that release chemical compounds that are naturally repellent or that mask their odour for antipathogen reasons [6]. Indeed, orang-utans occasionally cover their nest with branches from a different species having known mosquito-repellent activity (Largo et al., 2009, as cited in [10]). One preliminary study has shown that chimpanzees from the Toro-Semliki Wildlife Reserve in Uganda preferentially choose a tree that experimentally deter flying arthropods in the field [10].

Even, if the function and the duration of its use are different, some birds also used specific plants to build or add to their nest structure [11–14]. An antipathogen function was also suggested for their nest, where birds could use plant fragments with repellent or antimicrobial activity to help controlling pest and/or pathogen populations [11, 12, 15]. Some of these plants, like *Lavandula stoechas* or *Achillea ligustica* actually possess chemical compounds that are distilled and used by humans to make aromatic household cleaners, herbal medicines, and household disinfectants [14, 16]. Evidences of external use of medicinal plants by vertebrates occurred in other contexts like anointing behaviour in numerous primates species (*Cebus sp*. [17–19]; *Leontopithecus chrysomelas* [20]; *Pongo pygmaeus* [21]; and *Sapajus sp.* [17]). They have been observed topically applying plant material which contain secondary compounds like carvone, eugenol, linalool or apiole that are known to have anti-insect activity and/or medicinal benefits [18]. The anti-insect activity of a product, natural or chemical, can have different modes of action. The product can reduce vector–host contact: (i) a spatial repellent effect, i.e. odour causes a shifting of vectors away from the source; (ii) a contact irritant effect, i.e. insects move away after contact; (iii) an anti-feedant effect, i.e. blood feeding inhibition of female mosquitoes, and iv) toxic effect, i.e. a knock down and mortality effect [22, 23].

Humans have been taking advantage of these properties found in some plants to develop efficient products. Indeed, numerous studies report bioactive molecules from various plants, mostly small volatile and aromatic compounds that can be found in essential oils [24, 25]. For example, essential oil of *Cymbopogon winterianus*, *Cinnamomum zeylanicum* and *Thymus vulgaris* showed independently spatial repellent, contact irritant and toxic effect [26]. In addition, the process to obtain essential oil create a by-product, i.e. hydrolat, that have been proved to be valuable with bio-activities [27, 28].

In this study, first tree species in which wild chimpanzees of the Sebitoli community in Kibale National Park, Uganda, were investigated to see if there was selection. The Sebitoli habituated community belongs to the same population than two previously studied communities, where it was shown that at the height and altitude of their nests, mosquitoes were less abundant [8]. Further, if selection was confirmed, we examined whether this tree species selection could be explained by the potential insect deterrent effects of the leaves. Essential oils and hydrolats obtained from the hydro-distillation of leaves from trees selected by chimpanzees for nesting over other trees in their habitat were tested in behavioural assays (repellent, irritant and toxic) against an African mosquito, *Anopheles gambiae*. A further justification to the study is to use the information about chimpanzee tree nesting as a procedure to screen potential trees to identify those with repellent properties. This information could potentially be used to develop new repellents for use by humans to minimize contact with vector mosquitoes.

## Methods

### Study site—nesting behaviour

The study took place in the Sebitoli area located at the extreme North part of the Kibale National Park in Western Uganda (795 km^2^, 0°13′-0°41′N and 30°19′-30°32′E1 [29]). This Park is composed of a mid-altitude forest with high plant and animal diversity [30]. The climate of this equatorial area is composed of two rainy (from March to May, and September to November) and two dry seasons in between. The elevation is between 1110 and 1590 m, and the rainfall averages 1700 mm per year (https://ugandawildlife.org/https://ugandawildlife.org/, [31]). There is a gradient across the park marked by an increase in temperature and a decrease in rainfall from North to South [32]. The Sebitoli area, at the extreme Northern part of the park, thus characterized by lower temperature and higher rainfalls compared to other studied areas in the park. It is surrounded by many agricultural parcels such as small farms with food crops, tea and eucalyptus plantations. The Sebitoli forest is composed of 35% regeneration forest, 35% degraded forest, 14% mature forest and 14% terrestrial herbaceous vegetation [33]. The proportions of mature and regenerating forests are low compared to far Southern Ngogo chimpanzee territory, but quite similar to Kanyawara where the previous survey on mosquito and nest distribution was conducted [8]. Since 2009, the Sebitoli Chimpanzee Project team started a habituation process and monitored daily the chimpanzee (*Pan troglodytes schweinfurthii*) community that counts around 100 individuals, with 66 identified. Field assistants followed individuals daily, arriving at the nesting site before chimpanzees leave their nest to the time of nest construction. As soon as the chimpanzee habituation was sufficient to clearly identified individuals and follow them from nest to nest (2017), the identity of chimpanzees presents at the nesting site, the coordinates, the plant species used in the sleeping platform were recorded by the Sebitoli Chimpanzee Project team.

Data from June 2017 to June 2019 were analysed to determine the most frequent tree species used. A previous tree census from the same area, was conducted in 80 plots covering 26.3 ha in total. In order to be representative of the territory of the Sebitoli chimpanzees, the number and the locations of the plots in each type of vegetation were decided according to land-cover classes determined with satellite images (methods described in [34]). This survey recorded the occurrence of 95 tree species. To obtain the control tree species, the first ten species abundant but not or rarely used by chimpanzees (less than 1% of nest) were selected.

### Essential oils and hydrolats

Between 1 and 30 kg of fresh leaves were harvested from the 10 “nesting” tree species during the study period and the 10 “abundant” tree species. The tree species were collected in 2006 in Kibale National Park and determined at the herbarium of the Laboratoire de Phanérogamie at the Muséum National d’Histoire Naturelle (Paris, France) where voucher specimens have been deposited [35]. Two remained unknown despite attempted morphological identification and DNA sequencing. The leaves were air-dried and stored in a cool and dark place. Up to 500 g of shredded dry leaves were processed by hydrodistillation in a Clevenger apparatus (REUS, Contes, France) to extract the potential essential oils. To do this, the distillation chamber, filled with 6 to 8 L of water and the leaves, was heated until reaching a boiling state for three to four hours. The distillate was collected in a separating funnel in which the essential oil, if present was on top of the lower aqueous part known as the hydrolat. Essential oils and hydrolats were separately kept in a stoppered vial at 4 °C until there were tested for further mosquito bioassays. The yield of essential oil (%) is the ratio of the essential oil weight (g) divided per the dry leaves weight (100 g).

### Mosquitoes

Biological assays were conducted on female *An. gambiae* from the susceptible reference strain Kisumu reared at LIN-IRD in Montpellier, France. As a component of colony maintenance, the insecticide susceptibility of this strain was confirmed using World Health Organization (WHO) diagnostic concentrations (i.e. 4% DDT, 0.75% permethrin) and its genotypes for *kdr* and *ace*.1R mutations are controlled by PCR every 4 months. The colony is maintained in a climatic room at 27 ± 2 °C and 80 ± 10% relative humidity and with a photoperiod cycle of 12:12 h (light:dark). Larvae are fed with fish food and adults with 10% honey solution. Mosquito females used in the bioassays were 2 to 7 days old.

### Bioassays

Behavioural bioassays testing the activities of the essential oils and the hydrolats were done following and adapting the methods of a previous work (details and figures in [26]). For the essential oils with sufficient quantity, solutions were prepared, when possible, at 0.1 and 1% (volume/volume) diluted in a solvent constituted by 2/3 ethanol and 1/3 silicone oil (Dow CorningH 556 fluid). These concentrations were chosen after preliminary assays and based on published data [26]. For the 20 hydrolats, the solutions were not diluted and used at 100% concentration. To avoid any contamination, essential oil and hydrolats of only one plant were tested per day. The tests were conducted with papers being treated with a positive concentration gradient.

Three different assays were performed: spatial repellent, contact irritancy and toxicity. The minimum necessary quantity for the assays was uniformly deposited: 3.3 mL of a solution (essential oil with a given density of 0.9 g/mL or hydrolat) on 13 × 30 cm chromatography papers for spatial repellent assays, and 2 mL on 12 × 15 cm chromatography papers for contact irritancy and toxicity assays. Papers of the same sizes were also treated with 3.3 mL or 2 mL of solvent (ethanol-silicone or water) to be used as a negative control. All treated papers (three replicates each) were allowed to dry at room temperature for one hour before the test. To perform all mosquitoes’ assays at all concentrations with three replicates, more than 0.16 g of essential oil is needed. To perform all mosquitoes’ assays at 0.1% concentration of essential oil, at least 0.02 g is needed. When quantity was not enough for all assays at a given concentration, spatial repellency assays were prioritized.

### Spatial repellent assays

The high-throughput screening system (HTSS) used had two chambers of 30 × 10 cm [36]. Treated chromatograph papers, with products or with only the solvent, were rolled around the inner sur face of one chamber. In parallel, chromatograph papers without treatment were rolled around the inner surface of the other chamber. Thus, the two chambers, treated and untreated, were exposed to an equivalent ambient light. A polyethylene net of 0.3 mm mesh was inserted preventing direct mosquito contact with the treated paper. Two end caps covered both sides of the HTSS with a closable mosquito entry. Between the untreated and treated chambers, there was a ‘butterfly’ valve that allowed or not allowed mosquitoes to pass from a chamber to another. During assays, the HTSS was held steady and parallel to the bench top.

For each assay, around 20 female mosquitoes were transferred using mechanical aspiration into the treated chamber. After a 10 s acclimatization period, the butterfly valve was opened for 10 min. Mosquitoes moving from the treated to the untreated chamber were termed “escaped”. Conversely, mosquitoes remaining in the treated chamber were referred to as “stayed”. At the end of the test, the butterfly valve was closed and the number of mosquitoes “escaped” and “stayed” were recorded after a CO_2_ anesthesia. Tests for a given product were considered valid when less than 20% of “escaped” mosquitoes in the three control replicates. The ability of a plant extract to repel mosquitoes was estimated by the proportion of “escaped” mosquitoes: the higher (combining all replicates), the stronger the spatial repellency effect.

### Contact irritancy assays

These assays were performed with standard WHO test kit [37] with two tubes of 12 × 4 cm separated by a slide unit. The inner surface of a tube is covered with a treated chromatograph paper (solution or solvent), when the other tube with an untreated chromatograph paper. The mosquitoes are in direct contact with the papers.

For each test, around 20 female mosquitoes were initially placed inside the treated tube through the small hole in the slide unit. Then, the untreated tube was attached to the opposite side of the apparatus. After a 10 s acclimatization period, the slide unit was opened for 10 min, allowing the mosquitoes to move freely from one tube to the other. Mosquitoes moving from the treated to the untreated tube were considered “escaped”. Conversely, mosquitoes that remained in the treated tube were referred as “stayed”. Once the slide valve was closed, the number of mosquitoes “escaped” and “stayed” in each tube was recorded. For each product, the tests were considered valid when the proportion of “escaped” mosquitoes in the three control replicates was less than 50%. The contact irritant activity of a product was estimated on the basis of the proportion of “escaped” mosquitoes for the three replicates, with high activity resulting in high proportions.

### Toxicity assays

Toxicity assays were performed using the previously cited standard WHO test kit used for the contact irritancy assay. After an acclimatisation period of 30 min, a mean of 25 female mosquitoes were exposed for 1 h to the product or solvent in the tested tube. The mosquitoes were then transferred to an untreated tube containing a 10% sucrose solution and maintained at 27 °C and 80% relative humidity. The number of dead and live *An. gambiae* was recorded after 24 h. The test was considered valid when there were less than 10% dead mosquitoes in the control (the three papers treated with the solvent). The toxic effect of each product was expressed as the proportion of dead mosquitoes.

### Data analysis

All statistical analyses were performed using R software, version 3.3.2 (R Core Team, 2016).

To analyse tree selectivity in detail, a chi-squared test of independence was used to compare the frequencies of tree species used by chimpanzees for nesting with their recorded occurrence in the plots. Then, post-hoc comparisons with the standardized residuals were performed [38]. When the absolute value of standardized residuals is larger than 1.96, the observed frequency of the species is significantly different from the expected value at a probability level of 0.05. The tree species is considered as being selected and thus “preferred” (> 1.96) or “disfavoured” (< −1.96), the species can also be “indifferent” (−1.96 < standardized residuals < 1.96) according to their occurrence in the habitat. In the following analyses, the species most frequently used for nesting will be named “nesting” trees, and compared to “abundant” trees.

The proportions of escaped or dead mosquitoes in control and treated assays were compared using Fisher’s exact test by pooling the replicates. The proportions of escaped or dead mosquitoes were corrected by the control assay values using Abbot’s formula [39]. To compare the properties of the two datasets of trees (nesting vs abundant and preferred vs disfavoured), Fisher’s exact test adapted to small sample size was used on the proportions of species showing a significant activity for at least one solution.

## Results

### Nesting preference

**Fig. 1:**
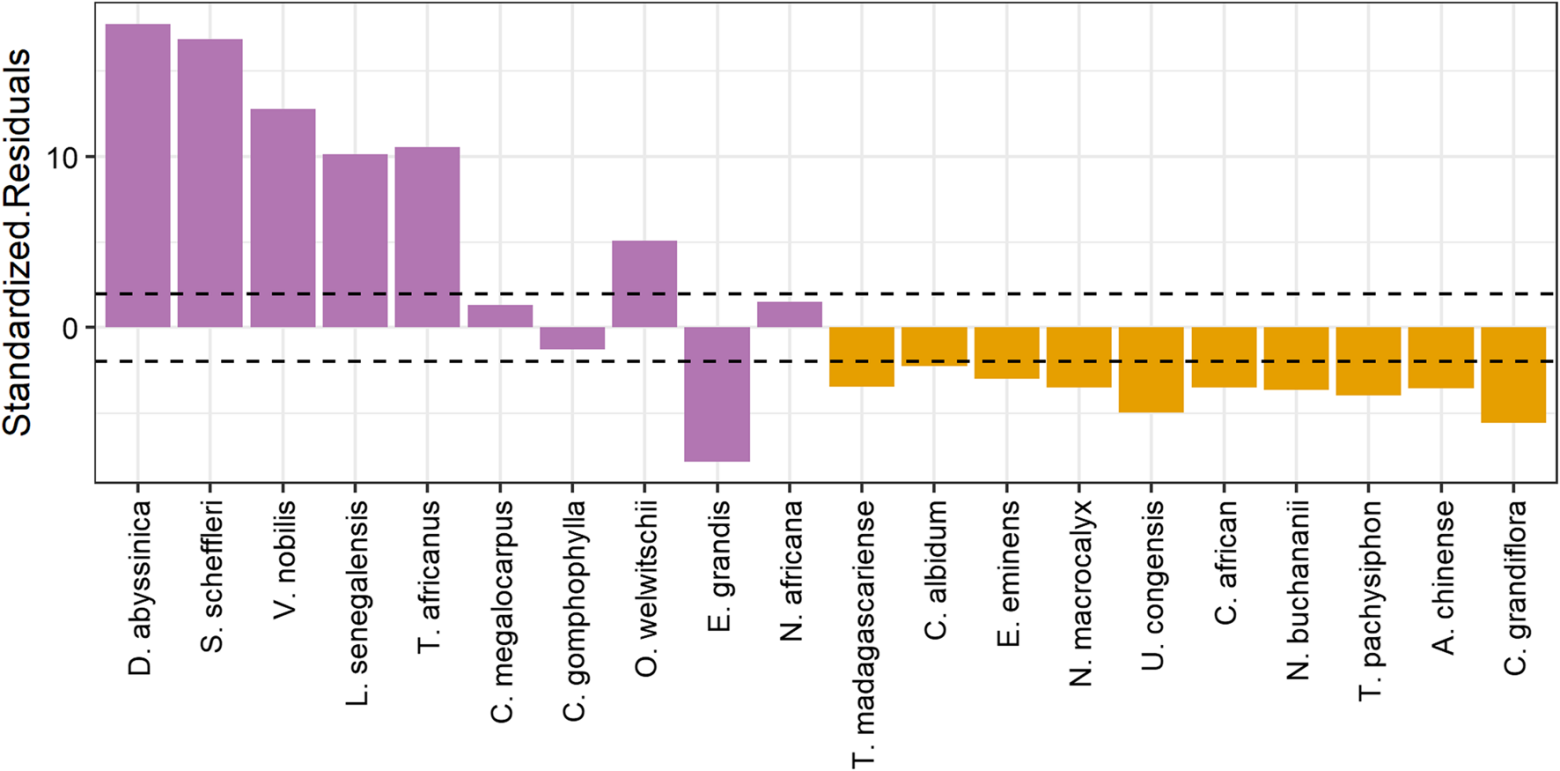
Nest tree selectivity. The standardized residuals from Chi-squared test: if they are over the top dashed line, the species are preferred, below the down dashed line, they are disfavoured compared to their occurrence in the habitat. In purple, the nesting species, in orange the abundant species

Between 2017 and 2019, 1081 nests of 41 chimpanzees were described corresponding to 425 nights. They were built in 42 tree species of which 10 accounted for more than 80% of the recorded nesting trees, when they represented only 30% of the Sebitoli habitat (Table 1). More precisely, *Diospyros abyssinica, Strombosia scheffleri, Vepris nobilis, Lepisanthes senegalensis, Turraeanthus africanus* and *Olea welwitschii* were preferred by chimpanzees according to their relative abundance. However, *Eucalyptus grandis* was disfavoured for nesting when taking into account their occurrence in the habitat (Fig. 1), chimpanzees were considered indifferent to *Croton megalocarpus, Celtis gomphophylla* and *Noronhia africana* for nesting when considering their natural occurrence.

**Table 1:** Nesting use, natural occurrence and essential oil collected from the 20 species investigated

Family	Species	Used for nesting (%)	Presence in habitat (%)^a^	Essential oil collected (g)	Yield (W/W%)
Nesting trees					
Ebenaceae	*Diospyros abyssinica*	27.84 (n = 255)	7.33 (n = 242)	0.1742^b^	0.0348
Olacaceae	*Strombosia scheffleri*	18.67 (n = 171)	3.42 (n = 113)	0.1526^c^	0.0305
Rutaceae	*Vepris nobilis*	8.62 (n = 79)	1.09 (n = 36)	0.2064^b^	0.0413
Sapindaceae	*Lepisanthes senegalensis*	6.99 (n = 64)	1.27 (n = 42)	0.2444^b^	0.0489
Meliaceae	*Turraeanthus africanus*	5.79 (n = 53)	0.27 (n = 9)	0.5223^b^	0.1045
Euphorbiaceae	*Croton megalocarpus*	4.37 (n = 40)	3.58 (n = 118)	0.3408^b^	0.0682
Cannabaceae	*Celtis gomphophylla*	3.17 (n = 29)	4.27 (n = 141)	0.0293^c^	0.0059
Oleaceae	*Olea welwitschii*	2.29 (n = 21)	0.55 (n = 18)	< 0.001	NA
Myrtaceae	*Eucalyptus grandis*	1.97 (n = 18)	10.39 (n = 343)	2.7961^b^	0.5592
Oleaceae	*Noronhia africana*	1.75 (n = 16)	1.18 (n = 39)	0.0193^d^	0.0039
Abundant trees					
Moraceae	*Trilepisium madagascariense*	0.55 (n = 5)	2.42 (n = 80)	0.0052	0.0010
Sapotaceae	*Chrysophyllum albidum*	0.44 (n = 4)	1.39 (n = 46)	0.0056	0.0011
Annonaceae	*Uvariopsis congensis*	0.33 (n = 3)	3.42 (n = 113)	0.0615^c^	0.0123
Euphorbiaceae	*Neoboutonia macrocalyx*	0.33 (n = 3)	2.06 (n = 68)	0	0
Capparaceae	*Euadenia eminens*	0.33 (n = 3)	1.67 (n = 55)	0.0311^c^	0.0062
Apocynaceae	*Tabernaemontana pachysiphon*	0.11 (n = 1)	2.03 (n = 67)	< 0.001	NA
Leguminosae	*Newtonia buchananii*	0.11 (n = 1)	1.76 (n = 58)	0.0139	0.0028
Cannabaceae	*Celtis africana*	0.11 (n = 1)	1.64 (n = 54)	0.1375^c^	0.0275
Meliaceae	*Carapa grandiflora*	0.00	3.85 (n = 127)	0.1385^c^	0.0277
Cornaceae	*Alangium chinense*	0.00	1.36 (n = 45)	0.4828^b^	0.0966

^a^based on [34]

^b^sufficient quantity for all assays at 0.1% and 1%

^c^sufficient quantity for all assays at 0.1%

^d^sufficient quantity only for repellent assay at 0.1%

### Yield of the extraction

Out of the 20 trees (combining the 10 nesting trees and the 10 abundant trees but rarely or not used for nesting), 19 produced essential oils. Yields were low (less than 0.11 W/W%) for all of them at the exception of *Eucalyptus grandis* (2.80 W/W%). Only seven samples (five nesting trees and two abundant trees) had enough quantity for all mosquitoes’ assays at both concentrations (more than 0.16 g), 13 had enough quantity for the three mosquitoes’ assays at 0.1% solution (more than 0.02 g). *Noronhia africana* had only enough quantity for one of the three tests, i.e. spatial repellent assay at 0.1% (Table 1).

### Results of the bioassays

The detailed number of mosquitoes per test and per species are available in the Additional file 1. None of the hydrolats were active in all the bioassays performed, with the exception of the trees *Vepris nobilis* and *Celtis africana* showing a lightly toxic effect on *An. gambiae* (Table 2).

**Table 2:** Bioassays results with Abbot’s correction of the 20 tree species

Species	Product	Repellent test (> 20%)	Irritancy test (> 50%)	Toxicity test (> 10%)
Nesting trees				
* Diospyros abyssinica*	1%	**69.77**	**55.16**	−2.27
	0.1%	**33.20**	**71.39**	−2.04
	Hydrolat	3.39	5.01	−4.29
* Strombosia scheffleri*	1%	−0.15		
	0.1%	4.36	40.30	5.99
	Hydrolat	0.00	0.81	−0.04
* Vepris nobilis*	1%	**47.23**	**74.59**	8.10
	0.1%	16.95	**86.86**	−10.54
	Hydrolat	4.76	1.54	**10.21**
* Lepisanthes senegalensis*	1%	**40.05**	**70.00**	3.90
	0.1%	**21.15**	**61.46**	1.87
	Hydrolat	1.88	20.53	−2.44
* Turraeanthus africanus*	1%	**52.90**	**84.39**	2.01
	0.1%	1.66	44.43	−1.41
	Hydrolat	6.78	36.08	3.53
* Croton megalocarpus*	1%	**41.38**	**63.95**	0.00
	0.1%	−9.74	8.33	−8.20
	Hydrolat	0.00	16.19	3.65
* Celtis gomphophylla*	1%			
	0.1%	10.64	−3.75	−5.27
	Hydrolat	4.03	−4.63	0.00
* Olea welwitschii*	1%			
	0.1%			
	Hydrolat	0.17	15.02	−4.72
* Eucalyptus grandis*	1%	**35.00**	**100.00**	−3.63
	0.1%	**19.99**	45.14	0.00
	Hydrolat	3.30	11.48	0.37
* Noronhia africana*	1%			
	0.1%	**22.15**		
	Hydrolat	3.02	-9.83	2.90
Abundant trees				
* Trilepisium madagascariense*	1%			
	0.1%			
	Hydrolat	1.81	15.12	−0.52
* Chrysophyllum albidum*	1%			
	0.1%			
	Hydrolat	5.12	20.68	−5.48
* Uvariopsis congensis*	1%			
	0.1%	**30.20**	**69.71**	−0.49
	Hydrolat	−2.13	8.16	−3.39
* Neoboutonia macrocalyx*	1%			
	0.1%			
	Hydrolat	2.78	-2.08	5.59
* Eudenia eminens*	1%			
	0.1%	15.63	**56.97**	1.91
	Hydrolat	1.67	6.18	1.91
* Tabernaemontan pachysiphon*	1%			
	0.1%			
	Hydrolat	3.17	26.76	−0.12
* Newtonia buchananii*	1%			
	0.1%			
	Hydrolat	7.09	22.76	1.45
* Celtis africana*	1%	**22.41**		
	0.1%	**38.31**	**79.07**	5.82
	Hydrolat	−1.82	7.08	**11.11**
* Carapa grandiflora*	1%	**21.09**		
	0.1%	10.28	**62.68**	5.72
	Hydrolat	−1.44	11.75	1.25
* Alangium chinense*	1%	15.63	**52.09**	0.12
	0.1%	16.67	**93.84**	−1.59
	Hydrolat	7.60	3.74	2.78

Negative values indicate a lower activity compared to the control. In bold, result significantly different from the control and superior at the threshold

**Fig. 2:**
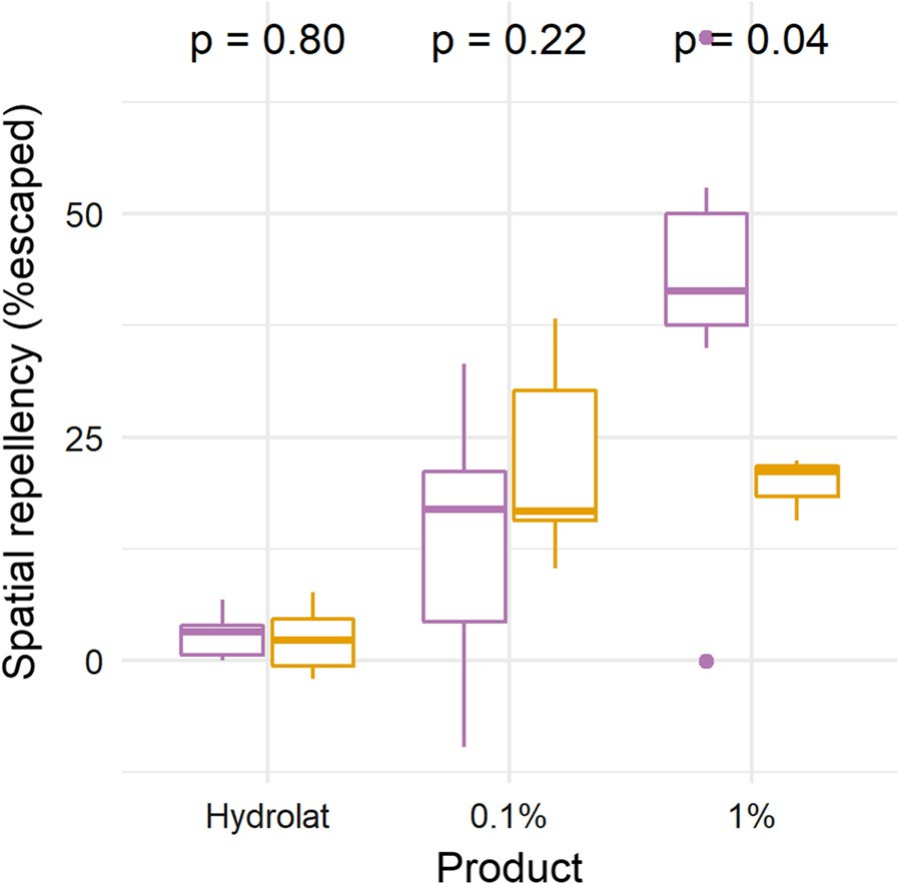
Boxplot of mosquitoes escaped percentage in spatial repellency assay per concentration according to tree category. In purple, the nesting trees, in orange the abundant species

Out of the nine nesting species tested, seven showed spatial repellent properties for at least one essential oil concentration: *Diospyros abyssinica, Lepisanthes senegalensis, Eucalyptus grandis* at both concentrations; *Vepris nobilis, Turraeanthus africanus, Croton megalocarpus* at 1%, and *Noronhia africana* at 0.1%. On the other hand, only three out of five abundant species tested showed spatial repellent property for at least one concentration: *Celtis africana* at both concentrations, *Carapa grandiflora* at 1% and *Uvariopsis congensis* at 0.1% (Table 1). At 1% concentration of the essential oils, nesting tree species are significantly more repellent than abundant trees but at 0.1% there is no significant difference of activities (Fig. 2).

So, 70% of spatial repellent essential oils detected in this study are from nesting species. The proportion of essential oil having spatial repellent properties for at least one essential oil concentration is greater for species used by chimpanzees compared to the abundant and not preferred tree species (66% vs 30%, Fisher’s test, X-squared = 3.200, df = 1, p-value = 0.0368). On the other hand, out of the six species preferred by chimpanzees (when taking into account their natural occurrence in the habitat), four showed repellent properties compared to four out of eleven species disvafoured by chimpanzees. The proportion of essential oil having repellent properties is not significantly different between preferred vs disfavoured tree species (0.67% vs 0.37%, Fisher’s test, X-squared = 1.431, df = 1, p-value = 0.2316).

All nine essential oils tested that showed spatial repellent activity for at least one essential oil concentration also demonstrated contact irritancy effect. In addition, the two abundant trees *Alangium chinense* and *Euadenia eminens* were also having irritant properties but they did not show spatial repellent activity. There is no difference of mosquitoes escaping in contact irritancy assays according to the category of tree per concentration. The proportion of essential oil tested having irritant properties for mosquitoes for at least one essential oil concentration is not significantly different between the nesting vs abundant trees (0.60% vs 0.50%, Fisher’s test, X-squared = 0.202, df = 1, p-value = 0.3265) and preferred vs not preferred trees (0.67% vs 0.55%, Fisher’s test, X-squared = 0.236, df = 1, p-value = 0.3137). No essential oils at any concentration showed toxic activity against *An. gambiae* in this study.

## Discussion

During the 425 nights of records, more than 80% of the 1081 nests were built in only ten tree species in the Sebitoli community. Six of them (i.e., *Diospyros abyssinica, Strombosia scheffleri, Vepris nobilis, Lepisanthes senegalensis, Turraeanthus africanus, Olea welwitschii*) were considered specifically selected by chimpanzees when taking into account their natural abundance in the habitat. Out of 20 species combining nesting and abundant trees species, 19 produced essential oils and 13 had enough volume to be tested in three mosquito behavioural assays. Tree species that have produced no or too little essential oil are considered to have little or no aromatic ability to repel mosquitoes. Interestingly, 70% of spatial repellent essential oils recorded in this study are from nesting species. At 1% concentration of essential oil, nesting tree species are significantly more repellent than abundant tree species. All essential oils showing some spatial repellent activity also showed contact irritancy effect against *An. gambiae*. However, no significant difference was observed when looking at contact irritancy property of essential oils issued of nesting species compared to abundant species, or preferred compared to disfavoured tree species. In addition, none of the essential oil appeared to be have insecticidal (toxicity) property. All the hydrolats showed no significant activities.

This study aimed to test one of the hypotheses behind nest tree selectivity, that chimpanzees might choose repellent tree species to avoid flying arthropods [10]. Although insectifuge volatile compounds are present in leaves of selected species and may partly explain chimpanzee selectivity on nesting trees, this does not fully explain all species selected. Indeed, the second most used tree for nesting, *Strombosia scheffleri* demonstrated no significant bio-activity. Among the preferred species, *Olea welwitschii* produced so little essential oil that it was not collected. Actually, some of the plant may have not enough volatile compounds in its leaves to act as repellent for chimpanzee. On the other hand, the abundant species *Uvariopsis congensis*, *Celtis africana* and *Carapa grandiflora* disfavoured by chimpanzees showed spatial repellency and contact irritancy effects.

This study tested only the activity of plants against one species of mosquito but product can be repellent to some species but less or not effective against other species [25]. Another limitation of this study is that only the repellency of the essential oil and hydrolat were tested, but it is possible that a plant has no repellent essential oil and is repellent though another channel. Moreover, this experiment was conducted on lab reared mosquitoes that may exhibit different behaviours compared to natural field populations.

A repellent tree species could create a “chemically” comfortable sleep by having fewer flying arthropods that can be considered a nuisance thought the frequency of the sound emitted [10]. That choice can also benefit chimpanzees indirectly: by avoiding mosquitoes bites, they can reduce the risk of encountering *Anopheles* potentially carrier of malaria parasites (ie *Plasmodium spp.*) and other parasites. In Kibale National Park, four different strains of *Plasmodium* (*Plasmodium reichenowi, Plasmodium vivax-like, Plasmodium billbrayi, Plasmodium billcollinsi*) were found in three wild chimpanzees sampled, confirming that chimpanzees carry mixed infections [40]. However, chimpanzees rarely display symptoms and the parasite load appears to be low. One possibility could be that individuals have been witnessed ingesting parts of medicinal plant with anti-malarial bioactivities [35, 47]. Malaria is not the only diseases that can be transmitted by mosquitoes, chimpanzees can also get infected by Chikungunya, Zika and West Nile virus [48]. Moreover, mosquitoes are not the only vectors, for example ticks can also carry and transmit arbovirus diseases (suspected Lyme Borreliosis in a captive chimpanzee [49] or the Kyasanur Forest Disease affecting monkeys in India [50]).

Both the repellent activities and the mechanical comfort could influence chimpanzees’ choice of the tree species [9] and it may also, probably be a trade-off between the two. Further studies investigating both aspects simultaneously should be considered to understand whether the choice is at the species level or in the repertoire is composed of some comfortable tree species and some repellent tree species and in which conditions they are selected. For example, a tree that is very comfortable but has no repellent activity could be chosen when the abundance of mosquito is low. This aspect was not studied here, but mosquitoes’ abundance and infection can vary across the year [41–46] and could be related to a differential use of repellent trees.

When investigating the existing literature on the 20 tree species studied [51], few studies have tested their repellent or pesticide properties. Interestingly, three nesting tree species and one abundant tree species have demonstrated repellent or insecticidal effect. *Diospyros abyssinica* leaf extracts show larvicidal activity against *Culex quinquefasciatus* and *An. gambiae* mosquitoes but not against *Aedes aegypti* [52]. The essential oil of *Eucalyptus grandis* leaves have shown repellent and toxic effect against another mosquito species, *Culex pipiens quinquefasciatus* [53]. *Turraeanthus africanus* expressed pesticidal capacity against the beetles *Sitophilus zeamais* and *Calusobruchus maculatus* [54]. Then, the hexane leaf extract of the abundant tree *Alangium chinense,* showed repellent or insecticidal property against the beetle *Tribolium castaneum* [55]. On the case of *Eucalyptus grandis*, it is also the only tree of the dataset not naturally occurring in Uganda. It was planted around the park for economical reason [56] and as a response to experiences of crop destruction by wildlife after serious degradation of the forest due to anthropogenic activities in the late 1960s and early 1970s [57] making it the most abundant tree in Sebitoli. This could explain why *Eucalyptus grandis* appeared in the 10 most used trees hosting nests while not being preferred by chimpanzees. This example highlights that the tree occurrence in the habitat should not be neglected when estimating tree selectivity in nesting behaviour. Interestingly, these findings partially corroborate the folk knowledge in Uganda where water or oil extract of *Eucalyptus grandis* leaves are used as pesticide and repellent [58]. Ethnoveterinary medicine records in Kenya reported that the decoction of *Vepris nobilis* and *Croton megalocarpus* are used to control and repel ticks in farm animals [59].

## Conclusion

This study highlighted promising new plants that, thanks to the knowledge of chimpanzees, pave the way for new bio-inspired solutions. Indeed, investigation on the composition of the different essential oils could provide valuable information on the molecules that may be responsible for the observed effects. Further trials are needed to test the essential oils at other concentration and against others vector species to validate and expand the project's prospects.

## Supplementary Information

**Additional file 1: Table S1.** Result of the spatial repellency assays for the 20 trees species. **Table S2.** Result of the contact irritancy assays for the 20 trees species. **Table S3.** Result of the toxicity assays for the 20 trees species.

## Data Availability

All data generated or analysed during this study are included in this published article and its supplementary information files.
