# Supplementary material for: Repellent activity against Anopheles gambiae of the leaves of nesting trees in the Sebitoli chimpanzee community of Kibale National Park, Uganda
Source: Malar J. 2022 Sep 27;21:271. doi: 10.1186/s12936-022-04291-7 (PMC9513939; doi:10.1186/s12936-022-04291-7)
Supplement: Supplementary file 1 — Additional file 1: Table S1. Result of the spatial repellency assays for the 20 trees species. Table S2. Result of the contact irritancy assays for the 20 trees species. Table S3. Result of the toxicity assays for the 20 trees species. [file 12936_2022_4291_MOESM1_ESM.docx]

**Supplementary 1. Plant information**

1. *Diospyros abyssinica* (Hiern) F.White

Synonyms: *Ebenus abyssinica* (Hiern) Kuntze, *Maba abyssinica* Hiern

*Diospyros abyssinica* is a species of trees in the Ebenaceae family that grows in Angola, Benin, Burkina, Cameroon, CAR, Chad, DRC, Eritrea, Ethiopia, Gabon, Ghana, Guinea, Ivory Coast, Kenya, Malawi, Mali, Mozambique, Nigeria, Sudan, Tanzania, Togo, Uganda, Zambia, Zimbabwe. It is a small, medium–sized or large tree up to 36 m in height that can be found in a wide diversity of forest types, and also in woodland and thickets, at elevations from 750 - 2,000 m.

2. *Strombosia scheffleri* Engl.

Synonyms: *Strombosia minor* Engl., *Strombosia toroensis* S.Moore

*Strombosia scheffleri* (Oleaceae) can be found in Angola, Burundi, Cameroon, Congo, DRC, Gabon, Kenya, Malawi, Mozambique, Nigeria, Rwanda, Sudan, Tanzania, Uganda, Zimbabwe. It is an evergreen tree that can reach up to 30 m, with low branches and a big crown. It is usually an understorey forest tree that ranges between 1200 and 2 500 m of altitude.

3. *Vepris nobilis* (Delile) Mziray

Synonyms: *Aspidostigma acuminatum* Hochst., *Crantzia nobilis* (Delile) Kuntze, *Teclea nobilis* Delile, *Teclea welwitschii* (Engl.) Verd., *Toddalia nobilis* (Delile) Hook.fil. ex Oliv.

*Vepris nobilis* (Rutaceae) is widely distributed in tropical Africa namely Burundi, Djibouti, DRC, Eritrea, Ethiopia, Kenya, Malawi, Rwanda, Somalia, Sudan, Tanzania, Uganda, Zambia, Zimbabwe and also, in Arabia. It is an evergreen shrub or, more commonly, a tree usually growing from 2 to 12 m high or even taller when growing in the rain-forest. It can be encountered in dry forest, thickets on rocky hills; wooded grassland; riverine formations; rain-forest; lake shores at elevations from 900 - 2,700 m.

4. *Lepisanthes senegalensis* (Poir.) Leenh.

Synonyms: *Aphania angustifolia* Radlk., *Aphania bifoliata* (Thwaites) Radlk., *Aphania boerlagei* Valeton*, Aphania cuspidata* (Blume) Radlk., *Aphania danura* (Roxb.) Radlk.*, Aphania dasypetala* Radlk.*, Aphania fascicularis* Radlk., *Aphania langsonensis* Gagnep.*, Aphania loheri* Radlk.*, Aphania longipes* Radlk.*, Aphania macrophylla* Radlk.*, Aphania masakapu* Melch., *Aphania montana Blume, Aphania nicobarica* Radlk., *Aphania ochnoides* Pierre ex Lecomte*, Aphania paucijuga* (Hiern) Radlk.*, Aphania philastreana* Pierre*, Aphania philippinensis* Radlk.*, Aphania rubra* (Roxb.) Radlk.*, Aphania senegalensis* (Juss. ex Poir.) Radlk.*, Aphania silvatica* A.Chev. ex Hutch. & Dalziel*, Aphania sphaerococca* Radlk.*, Aphania spirei* Lecomte*, Aphania viridis* Pierre*, Deinbollia claessensii* De Wild.*, Didymococcus danura* (Roxb.) Blume*, Dimocarpus verticillatus* Roxb. ex Wall.*, Euphoria attenuata* Planch.*, Euphoria attenuata* Planch. ex Hiern*, Euphoria danura* (Roxb.) Wall.*, Euphoria rubra* (Roxb.) Royle*, Euphoria verticillata* (Roxb.) Lindl*., Hydnocarpus tamianus* Pulle*, Lepisanthes senegalensis subsp. sylvatica* (A.Chev. ex Hutch. & Dalziel) Aubrév., *Nephelium bifoliatum* Thwaites*, Nephelium danura* Roxb. ex G.Don, *Nephelium rubrum* (Roxb.) Walp., 1846, *Nephelium verticillatum* (Roxb.) G.Don, *Ornitrophe thyrsoides* Schumach. & Thonn., *Otophora paucijuga* Hiern, *Sapindus abyssinicus* Fresen., *Sapindus attenuatus* Wall. ex Hiern, *Sapindus bifoliatus* (Thwaites) Hiern, *Sapindus cuspidatus* Blume, *Sapindus danura* (Roxb.) Voigt*, Sapindus microcarpus* (Wight & Arn.) Kurz*, Sapindus ruber* (Roxb.) Kurz*, Sapindus senegalensis* Juss. ex Poir.*, Sapindus verticillatus* (Roxb.) Kurz*, Schmidelia thyrsoides* (Schumach. & Thonn.) Baker*, Scytalia danura* Roxb.*, Scytalia rubia* Roxb.*, Scytalia verticillata* Roxb.

*Lepisanthes senegalensis* belongs to the Sapindaceae family and is widespread throughout tropical Africa, tropical Asia, New Guinea and northern Australia. It is an evergreen shrub or tree with a dense, spreading crown; it can grow up to 16 m tall. They are generally present in the humid sites of a forest, and in the forest and riparian areas of a savannah; at altitudes of up to 1,800 m.

5. *Turraeanthus africanus* (Welw. ex C.DC.) Pellegr.

Synonyms: *Bingeria africana* A.Chev., *Guarea africana* Welw. ex C.DC., *Turraeanthus africana* (Welw. ex C.DC.) Pellegr., *Turraeanthus malchairi* De Wild., *Turraeanthus vignei* Hutch. & Dalziel, *Turraeanthus zenkeri* Harms

*Turraeanthus africanus* is a tree belonging to the family of Meliaceae which grows in tropical and subtropical regions from Sierra Leone to the Congo and Angola. It grows up to 40m high, with heavy dark green foliage. It is usually found in dense stands in rain forests alongside lakes and streams.

6. *Croton megalocarpus* Hutch*.*

*Croton megalocarpus* (Euphorbiaceae) occurs in Burundi, DRC, Kenya, Malawi, Mozambique, Rwanda, Tanzania and Uganda. It is a medium-sized to fairly large tree up to 35 m tall with a dense, spreading, rather flat crown. It is found in evergreen and semi-deciduous forest at 700 to 2400 m altitude, sometimes also in riverine woodland and wooded grassland. It has gained interest as a commercial poultry feed and bio-fuel crop.

7. *Celtis gomphophylla* Baker

Synonyms: *Celtis dioica* S.Moore, *Celtis durandii* Engl., *Celtis ugandensis* Rendle, *Sponia integrifolia* Boivin, *Trema integrifolia* Baill.

*Celtis gomphophylla* belongs to the Cannabaceae family and is found in tropics and subtropics Africa: from Ivory Coast to Ethiopia, to Angola, Zambia, Zimbabwe, South Africa and Madagascar. It exists from a shrub to a tree with a spreading crown; from 3 - 30 m tall, or even up to 60 m. It can be evergreen or deciduous. It occurs in the understory of moist evergreen, semi-deciduous and riverine forest, often in secondary formations in central Africa; restricted to upland forest in west Africa; forest edges, thickets, woodland and wooded grassland in east Africa, coastal forest in south Africa. It can be found at elevations up to 1,750 m.

8. *Olea welwitschii* (Knobl.) Gilg & G.Schellenb.

Synonyms: *Linociera welwitschii* (Knobl.) Knobl., *Mayepea welwitschii* Knobl., *Olea capensis* subsp. *welwitschii* (Knobl.) Friis & P.S.Green, *Olea mussolinii* Chiov., *Osmanthus welwitschii* (Knobl.) Knobl., *Steganthus welwitschii* (Knobl.) Knobl.

*Olea welwitschii* (Oleaceae) is native to Angola, Ethiopia, Kenya, Malawi, Mozambique, Tanzania, Uganda, Zambia, Zimbabwe. It is a tall tree, generally 12–24 m in height, with a straight, less branched trunk and a rather small crown. It can be found in lowland rain to upland dry evergreen forest around 750–1950 m.

9. *Eucalyptus grandis* W.Hill ex Maiden

*Eucalyptus grandis* (Myrtaceae) is a plant native of the humid subtropical and tropical regions of eastern Australia. It is an evergreen tree with a wide-spreading, rather thin crown; usually growing 40-55 m tall but exceptionally to 75 m.

10. *Noronhia africana* (Knobl.) Hong-Wa & Besnard

Synonyms: *Chionanthus africanus* (Knobl.) Stearn, *Linociera fricana* (Knobl.) Knobl., *Linociera angolensis* Baker, *Linociera dasyantha* Gilg & G.Schellenb., *Linociera fragrans* Gilg & G.Schellenb., *Linociera giordanii* Chiov., *Linociera johnsonii* Baker, *Linociera latipetala* M.Taylor, *Linociera mildbraedii* Gilg & G.Schellenb., *Linociera oreophila* Gilg & G.Schellenb., *Mayepea fricana* Knobl., *Olea mildbraedii* var. fricana Knobl., *Olea mildbraedii* var. lanceolata Knobl.

*Noronhia africana* belongs to the Oleaceae family and is found in Angola, Cameroon, CAR, DRC, Ghana, Guinea, Ivory Coast, Liberia, Nigeria, Sierra Leone, Tanzania, Uganda. It is a small to medium-sized tree or bush, 3 to 20 m high, with a rather twisted trunk with a circumference of 1 m, bearing a wide spreading crown. It can be found in lowland to upland rain forest around 1000–1200 m.

11. *Carapa grandiflora* Sprague

*Carapa grandiflora* belongs to the Sapindaceae family and is native to Burundi, DRC, Rwanda, Tanzania, Uganda. It is a tree up to 25 m, with short stem and wide spreading crown.

12. *Uvariopsis congensis* Robyns & Ghesq.

*Uvariopsis congensis* (Annonaceae) is native to Angola, Cameroon, CAR, DRC, Gabon, Ivory Coast, Kenya, Sudan, Uganda, Zambia. It is an evergreen shrub or a small tree with spreading branches; it can grow 4 - 12 m tall. It is an understorey plant of fringing forest and forest margins, also in secondary evergreen forest; at elevations from 1,080 - 1,650 m.

13. *Trilepisium madagascariense* DC.

Synonyms: *Bosqueia angolensis* Ficalho, *Bosqueia boiviniana* Cord. ex Baill., *Bosqueia calcicola* Leandri*, Bosqueia carvalhoana* Engl., *Bosqueia cerasiflora* Volkens ex Engl., *Bosqueia danguyana* Leandri, *Bosqueia gymnandra* Baker, *Bosqueia manongarivensis* Leandri, *Bosqueia occidentalis* Leandri, *Bosqueia orientalis* Leandri, *Bosqueia phoberos* Baill., *Bosqueia spinosa* Engl., *Bosqueia thouarsiana* Cord. ex Baill., *Bosquiea welwitschii* Engl., *Centrogyne angolensis* Welw. ex Benth. & Hook.fil., *Pontya excelsa* A.Chev., *Trilepisium gymnandrum* (Baker) J.Gerlach

*Trilepisium madagascariense* (Moraceae) is native to tropical and subtropical West and Central Africa, and occurs southwards to Zimbabwe, Mozambique, and South Africa. It grows to a medium-sized deciduous tree of up to 30 m height with drooping branches and a small, rounded crown. It grows in evergreen and semi-deciduous forests, flooded forests or forest patches and often grows along rivers and streams, extending on to the borders of savanna. It is found at altitudes of up to 2,000 m and higher.

14. *Neoboutonia macrocalyx* Pax

*Neoboutonia macrocalyx* (Euphorbiaceae) is found in Burundi, Cameroon, DRC, Kenya, Malawi, Rwanda, Tanzania, Uganda, Zambia, Zimbabwe. It is an open-crowned tree 10–20(40) m in height. It occurs mainly in medium altitude tropical rain forests at 600–2500 m, as a pioneer species in clearings and along streams. Secondary forests can consist of almost pure stands.

15. *Tabernaemontana pachysiphon* Stapf

Synonyms: *Conopharyngia angolensis* (Stapf) Stapf, *Conopharyngia cumminsii* Stapf, *Conopharyngia holstii* (K.Schum.) Stapf, *Conopharyngia pachysiphon* (Stapf) Stapf, *Sarcopharyngia angolensis* (Stapf) L.Allorge, *Tabernaemontana angolensis* Stapf, *Tabernaemontana holstii* K.Schum., *Tabernaemontana pachysiphon var. cumminsii* (Stapf) H.Huber*, Voacanga dichotoma* K.Schum.

*Tabernaemontana pachysiphon* (Apocynaceae) is occurring in the tropical Africa. It is an evergreen shrub or medium-sized tree growing from 2 to 18 m tall. It is commonly found in light forest understorey and riverine forests, from sea level to 1,500 m.

16. *Newtonia buchananii* (Baker) G.C.C.Gilbert & Boutique

Synonyms: *Piptadenia buchananii* Baker, *Piptadenia lujae* De Wild., *Piptadenia schweinfurthii* Vatke ex Engl.

*Newtonia buchananii* is geographically distributed from Nigeria, eastwards to Kenya, and south to Angola, Cameroon, DRC, Uganda, Tanzania, Malawi, Zambia, Zimbabwe, and Mozambique. It is described as a deciduous tree with a flat-topped crown and wide-spreading branches; it can grow from 10 to 40 m tall. This tree is found in highland forest areas at elevations from 1,100 - 1,800 m in west Africa. In East and southern Africa, it occurs in evergreen rainforest, often along watercourses and lakes, at elevations from 600 - 2,200 m.

17. *Euadenia eminens* Hook.fil.

Synonyms: *Crateva eminens* (Hook.fil.) Christenh. & Byng, *Euadenia alimensis* Hua, *Euadenia major* Hua, *Euadenia pulcherrima* Gilg & Gilg-Ben., *Ritchiea dolichocarpa* Gilg & Gilg-Ben.

*Euadenia eminens* (Capparaceae) is native to Cameroon, CAR, Congo, DRC, Gabon, Ghana, Guinea, Ivory Coast, Liberia, Sierra Leone, Uganda. It is a shrub or small tree up to 5 m. It can be found at elevations from1300 to 1650 m.

18. *Celtis africana* Burm.fil.

Synonyms: *Celtis burmannii* Planch., *Celtis eriantha* E.Mey. ex Planch., *Celtis henriquezii* Engl., *Celtis holtzii* Engl., *Celtis kraussiana* Bernh., *Celtis opegrapha* Planch., *Celtis rhamnifolia* C.Presl, *Celtis vesiculosa* Hochst. ex Planch.

*Celtis africana* Burm.f. (Ulmaceae) is common and widespread in South Africa, South west Arabian Peninsula, Comoros and Madagascar. It is a medium-sized tree that grows up to 30 m tall; the foliage is dark green. It occurs in a wide range of habitats usually found in the deciduous forests.

19. *Chrysophyllum albidum* G.Don

Synonyms: *Achras sericea* Schumach. & Thonn., *Chrysophyllum henriquesii* Engl., *Chrysophyllum kayei* S.Moore, *Chrysophyllum millenianum* Engl., *Gambeya albida* (G.Don) Aubrév. & Pellegr., *Planchonella albida* (G.Don) Baehni

*Chrysophyllum albidum* belongs to the Sapotaceae family and is common throughout the tropical Central, East, and West Africa regions. It is primarily a lowland rain forest tree species that grows up to 25 to 37 m in height at maturity.

20. *Alangium chinense* (Lour.) Harms

Synonyms: *Alangium begoniifolium* (Roxb.) Baill., *Alangium kenyense* Chiov*., Alangium octopetalum* Hanes ex Blanco, *Alangium platanifolium* f. *triangulare* Wangerin, *Guettarda jasminiflora* Blanco, *Karangolum chinense* (Lour.) Kuntze, *Marlea affinis* Decne., *Marlea begoniifolia* Roxb., *Marlea chinensis* (Lour.) Druce, *Marlea virgata* Zoll., *Stelanthes solitarius* Stokes, *Stylidium bauthas* Lour. ex B.A.Gomes, *Stylidium begoniifolium* (Roxb.) Voigt, *Stylidium chinense* Lour., *Stylis chinensis* (Lour.) Poir.

*Alangium chinense* (Lour.) Harms. (Alangiaceae) is widely distributed through East tropical Africa and in East Asia. It is an evergreen tree growing up to 24 m tall. A pioneer species in partly cleared areas of lowland and upland rain-forest.
